# The strength of coughing may forecast the likelihood of spread of multi-drug resistant microorganisms from the respiratory tract of colonized patients

**DOI:** 10.1186/s13756-014-0038-z

**Published:** 2014-12-12

**Authors:** Magda Diab-Elschahawi, Luigi Segagni Lusignani, Peter Starzengruber, Dieter Mitteregger, Andrea Wagner, Ojan Assadian, Elisabeth Presterl

**Affiliations:** Department of Hospital Hygiene and Infection Control, Vienna General Hospital, Medical University Vienna, Waehringer Guertel 18-20, 1090 Vienna, Austria; Division of Clinical Microbiology, Department of Laboratory Medicine, Medical University Vienna, Vienna, Austria

**Keywords:** Multidrug resistant microorganism, Cough, Aerogene spread, Risk factors, Single room isolation, Infection Control

## Abstract

**Background:**

Current recommendations indicate that patients who are coughing and have multidrug resistant microorganisms (MDROs) in their sputum are considered to be shedders and should be cared for in single room isolation at least until symptoms resolve. Airborne spread and subsequent contamination of surfaces adjacent to patients may contribute to transmission. Hence, isolation measures for patients colonized or infected with MDRO at their respiratory tract are intended to interrupt such transmission. However, the potential for microbial shedding in patients with MDRO-positive microbiological reports from their respiratory tract and factors justifying the need for single room isolation are viewed controversially.

**Methods:**

Cough aerosol produced by patients colonized with MDROs was measured for viable counts. Descriptive analysis together with logistic regression analysis was performed to assess the impact of strength of cough on growth of MDRO on culture plates.

**Results:**

In 18% (23/128) MDRO were transmitted. Multivariate analysis revealed that strength of cough significantly predicts the yield of MDRO on culture plates (P = 0.012).

**Conclusion:**

Based on these results it can be concluded that risk stratification for decision of single room isolation of patients colonized or infected with MDROs at their respiratory tract may also take the severity of cough into consideration. However, more work is required in order to assess the severity of cough objectively.

## Background

Multidrug resistant microorganisms (MDROs) may be transmitted by different routes, including blood borne, droplet, airborne and contact transmission. In general, and for all health care settings, adherence to hand hygiene practices may have the highest impact on prevention of direct and indirect contact transmission [1]. However, airborne spread and subsequent contamination of surfaces adjacent to patients may also contribute to transmission [2-5]. Isolation measures for patients colonized or infected with MDRO at their respiratory tract are intended to interrupt such transmission. Current recommendations [6,7] indicate that patients who are coughing and have MDRO in their sputum are considered to be shedders and should be cared for in single room isolation at least until symptoms resolved. However, in many healthcare facilities the availability of single room isolation is limited, and the automatic and prolonged use has been questioned [8]. Information on the ability of spreading MDRO through coughing is scant and risk factors to identify those patients who shall be considered as relevant aerogene shedders of MDRO are unknown.

The aim of this study was to determine the potential for microbial shedding in patients with an MDRO-positive microbiological report from their respiratory tract and to identify factors justifying the need for single room isolation.

## Methods

### Inclusion criteria and specimen collection

During a 1- year period all patients older than 18 years admitted to the University hospital of Vienna (VUH) whose respiratory tract was found to be colonized or infected with methicillin-resistant *Staphylococcus aureus* (MRSA), extended-spectrum beta-lactamase producing gram-negative bacteria (ESBL) including *Escherichia coli* or *Klebsiella pneumoniae*, or vancomycin resistant enterococci (VRE) were included. Subjects were asked to cough onto two culture plates after taking a maximal inspiration, i.e. Columbia blood agar with 5% sheep blood (Becton Dickinson GmbH, Heidelberg, Germany) and depending on the nature of the colonizing organism a selective agar plate: chromID® *S. aureus*, chromID® ESBL, chromID® VRE, bioMérieux, Marcy l’Etoile, France). For all samples, the same infection control practitioner positioned culture plates 5 cm in front of the patient’s mouth. The strength of cough was denoted as “strong (++)” or “weak (+). Participants gave written informed consent. The Ethics Review Committee of The Medical University of Vienna approved the study (EC No. 1140/2012).

Specimens were labelled and immediately transferred to the microbiology laboratory for further analysis. Culture plates from all specimens were incubated at 37°C and examined after 24 and 48 h. All possibly significant isolates were identified to species level using the specific colony coloration of the chromogenic medium while in case of growth limited to the blood agar plate or in case of ambiguous green-coloured colonies on the chromID® ESBL by MALDI-TOF mass spectrometry (Bruker Daltonik GmbH, Bremen, Germany). Appropriate resistance testing according to current EUCAST recommendations (www.eucast.org) was performed in order to confirm the drug resistance status.

### Statistical analysis

Statistical analysis was performed using SPSS software, version 20.0. Beyond descriptive analysis, a logistic regression analysis was performed to assess the impact of the independent variables (age, gender, species of MDRO, strength of cough) on the dependent variable (growth of MDRO on culture plate) using 95% CI and adjusted odds ratio (AOR). A P-value of less than 0.05 was considered to be statistically significant.

## Results and discussion

A total of 128 cough episodes were analysed. Demographic data and clinical information are summarized in Table 1. In total, 18% (23/128) of patients colonized or infected with MDRO transmitted the organisms from their respiratory tract onto the culture plates by coughing. Multivariate logistic regression analysis (Table 2) revealed that presence or absence of MDRO on culture plates was significantly associated with strength of cough (P = 0.012), but not with patients’ age (P = 0.593) or gender (P = 0.148), or the microbial species of the investigated MDRO (P = 0.523).

**Table 1:** **Demographic data and clinical characteristics of the study population**

	**Cough plates- outcome (growth/no growth)**		**Total n = 128**
**Variables**	**growth**	**no growth**	**n (%)**
	**(n = 23)**	**(n = 105)**	
Gender			
Male	14	52	66 (51.6)
Female	9	53	62 (48.4)
Age			
*Median (IQR)*	*66 (53-72)* years		
≤65 years	13	50	63 (49.2)
>65 years	10	55	65 (50.8)
Microorganism			
MRSA	10	41	51 (39.8)
VRE	4	31	35 (27.3)
ESBL	9	33	42 (32.8)
Strength of cough			
Strong	21	67	88 (68.8)
Weak	2	38	40 (31.3)

**Table 2:** **Multivariate logistic regression analysis of growth of MDRO as dependent variable**

	**MDRO growth on agar-plate (yes/no)**		
**Independent variables**	**Adjusted odds ratio (AOR)**	**95% CI**	**P-value**
**Age**	1.295	0.501-3.346	0.593
**Gender**	2.038	0.776-5.353	0.148
**Any Species of MDRO**	0.834	0.477-1.457	0.523
**Strength of cough**	7.336	1.558-34.537	**0.012**

*P-* value calculated by multiple logistic regression analysis.

Retrieval or absence of MRSA on culture plates as compared to other MDROs was not statistically significant (P = 0.955), however, the strength of coughing correlated significantly with MRSA retrieval (P = 0.014). The same observation was found for VRE (P = 0.275 and P = 0.015, respectively), and ESBL (P = 0.266 and P = 0.010, respectively). The stratified subset analyses for the investigated MDROs confirmed the observation made for MDROs in general, supporting the result that strength of coughing, but not the respective species, is associated with shedding or non-shedding.

In our institution, the current practice for single room isolation of patients colonized or infected with MDRO is based on a structured risk assessment strategy. Depending on an individual assessment which considers the possibility for transmitting microorganisms on basis of their anatomic location, patients may fall into one of three categories: (a) no isolation, (b) contact isolation or (c) strict isolation in a single room. For patients colonized with MDRO at their respiratory tract, as indicated by positive microbiological yield from sputum, tracheal secret, pharynx or anterior nares, single room isolation will be required, provided the patient is not ventilated with a closed breathing circuit. So far, there is little data available to decide if such patients may be epidemiologically relevant aerogene shedders of MDROs, since the role of droplet transmission in MDRO spread is not sufficiently investigated [5,7,9]. However, although this approached may be proactive and provides a safe environment of care for other patients, the benefits of single room isolation of patients colonized in their respiratory tract or their cohorting for reducing the spread of MDRO are discussed controversially [8]. Wigglesworth et al. [10] concluded that either isolation capacity needs to be increased or evidence-based risk assessment shall be applied in situations where isolation demands exceed availability.

Our study demonstrates that about every fifth patient colonized in the respiratory tract will transfer viable MDROs to close surfaces during coughing. However, a multivariate analysis reviles that transmission is seven times (AOR: 7.33) more likely if the patients coughs strongly. Based on these results it could be suggested that strict single room isolation may not be necessary in all patients colonized or infected with MRDOs at their respiratory tract. Aside of the anatomic location of microorganisms, the patient’s compliance to follow standard infection control measures, and his mobility, the severity of cough may potentially be a further aspect which could be used for an individual risk-assessment in order to decide for a single room isolation.

Our study has a number of limitations. The assessment of cough intensity was based on subjective judgement and differentiated only “strong” and “weak”. Although the same infection control practitioner assessed all cough intensities, and misclassification of cough intensities due to different observers was reduced to a minimum, a standardized method of measurement allowing accurate interpretation would be an advantage for future studies. A second limitation is that environmental surfaces > 5 cm from the patient were not sampled in parallel for presence or absence of MDRO. Therefore, the presented results do not allow statements on how far MDROs can be spread when a patient is coughing.

## Conclusion

Based on the present results it can be concluded that risk stratification for decision of single room isolation of patients colonized or infected with MDROs at their respiratory tract may also take the severity of cough into consideration. However, more work is required in order to assess the severity of cough objectively.

## Abbreviations

AOR: Adjusted odds ration
CI: Confidence Interval
ESBL: Extended-spectrum beta-lactamase
EUCAST: European Committee on Antimicrobial Susceptibility Testing
MALDI-TOF: Matrix-assisted laser desorption localization - time-of-flight
MDRO: Multidrug resistant microorganism
MRSA: Methicillin-resistant *Staphylococcus aureus*
VRE: Vancomycin resistant enterococci
